# Familial Transmission and Phenotypic Heterogeneity in Germline GATOR1-Related Epilepsy: A Systematic Review and Meta-Analysis

**DOI:** 10.3390/ijms27219773

**Published:** 2026-10-30

**Authors:** Lingxue Li, Yawen Bao, Lihan Huo, Xinna Ji, Qian Chen

**Affiliations:** 1 Capital Institute of Pediatrics, Chinese Academy of Medical Sciences & Peking Union Medical College, Beijing 100020, China; b2024032013@student.pumc.edu.cn; 2 Department of Neurology, Capital Center for Children’s Health, Capital Medical University, Capital Institute of Pediatrics, Beijing 100020, China; b184717616@163.com (Y.B.); m19832114833@163.com (L.H.); 3 Capital Institute of Pediatrics–Peking University Teaching Hospital, Capital Center for Children’s Health, Capital Medical University, Beijing 100020, China

**Keywords:** GATOR1, genotype–phenotype correlation, variable expressivity, familial transmission, somatic second hit, epilepsy

## Abstract

Incomplete penetrance and variable expressivity complicate interpretation of germline GATOR1 variants and individual-level phenotype prediction. We conducted a family-based systematic review and meta-analysis, reconstructing individual-level data from published pedigrees and reassessing variants using GATOR1-specific classification criteria. Sixty-three studies were included; 59 contributed 734 individuals heterozygous for germline pathogenic or likely pathogenic (P/LP) variants from 227 families. The pooled proportion of affected individuals was 65.7%. Recurrent variant status, variant consequence, *DEPDC5* protein domain, transmitting-parent phenotype, and overall parental origin did not consistently distinguish affected from unaffected individuals, although paternal *DEPDC5* transmission showed an exploratory association with higher disease expression (90.4% vs. 82.4%, *p* = 0.032). Among affected individuals, missense variants were associated with earlier seizure onset than loss-of-function (LoF) variants (0.67 vs. 4.50 years, *p* = 0.002). LoF variants in the DEPDC5 LassoA domain showed exploratory associations with cognitive impairment, MRI abnormalities, and epilepsy surgery (FDR-adjusted *p* ≤ 0.046). An expanded synthesis identified 14 published same-gene germline–somatic second-hit cases, of which 85.7% had focal cortical dysplasia (FCD) IIa; quantifiable somatic variant allele fractions (VAFs) for sequence-variant second hits were below 5% in all six single-region cases, whereas multiregion sampling in three cases showed spatial variation, including values below 1%. These findings suggest that disease expression and post-onset phenotype represent partly distinct aspects of clinical heterogeneity in GATOR1-related epilepsy.

## 1. Introduction

Epilepsy is a common neurologic disorder, with genetic factors estimated to contribute to approximately 30% of cases [1]. In familial focal epilepsy, genes encoding components of the GATOR1 complex, including *DEPDC5*, *NPRL2*, and *NPRL3*, represent important genetic causes [2,3,4]. GATOR1 functions within the amino acid-sensing pathway and negatively regulates mechanistic target of rapamycin complex 1 (mTORC1) signaling via Rag GTPases, thereby influencing neuronal growth, cortical development, and neuronal excitability [5,6,7]. Incomplete penetrance and variable expressivity are defining features of GATOR1-related epilepsy: individuals carrying the same familial variant may remain unaffected or develop epilepsy with markedly different clinical manifestations [8,9,10]. This distinction is clinically relevant to genetic counseling because penetrance estimates quantify the likelihood of disease expression but do not capture the range of phenotypes that may follow once epilepsy develops.

One potential biological explanation for this heterogeneity is a two-hit mechanism. Human brain-tissue studies have shown that a germline GATOR1 variant may establish genetic susceptibility, whereas an additional somatic event in a restricted population of brain cells can produce focal biallelic disruption, mTORC1 activation, and cortical pathology [11,12]. However, the contribution of brain somatic second hits remains difficult to quantify because tissue is available primarily from affected individuals undergoing epilepsy surgery, while low-level or spatially restricted mosaicism may escape detection [11,12,13].

A recent *DEPDC5*-specific synthesis quantified penetrance and systematically characterized phenotypic and treatment outcomes [14]. Across the GATOR1 complex, however, whether germline and familial characteristics associated with disease expression are similarly associated with post-onset phenotype remains unclear. Estimates derived from published pedigrees may also be influenced by proband-centered ascertainment and incomplete evaluation of unaffected relatives [15,16]. We conducted a family-based systematic review and meta-analysis of *DEPDC5*, *NPRL2*, and *NPRL3*, reconstructing individual-level genotype–phenotype and transmission data and re-evaluating reported variants using GATOR1-specific classification criteria [10]. The primary analysis was restricted to individuals heterozygous for germline pathogenic or likely pathogenic (P/LP) variants, with disease expression and post-onset phenotype evaluated separately. Matched parent–offspring transmissions enabled direct assessment of transmitting-parent phenotype and parental origin, while germline variant characteristics and brain somatic second-hit evidence provided complementary genetic and tissue-level perspectives. This study aimed to determine whether familial and germline genetic factors show distinct associations with disease expression and post-onset phenotype, while characterizing brain somatic mosaicism as an additional tissue-specific component of phenotypic heterogeneity.

## 2. Methods

### 2.1. Study Design, Registration, and Search Strategy

**Figure 1:**
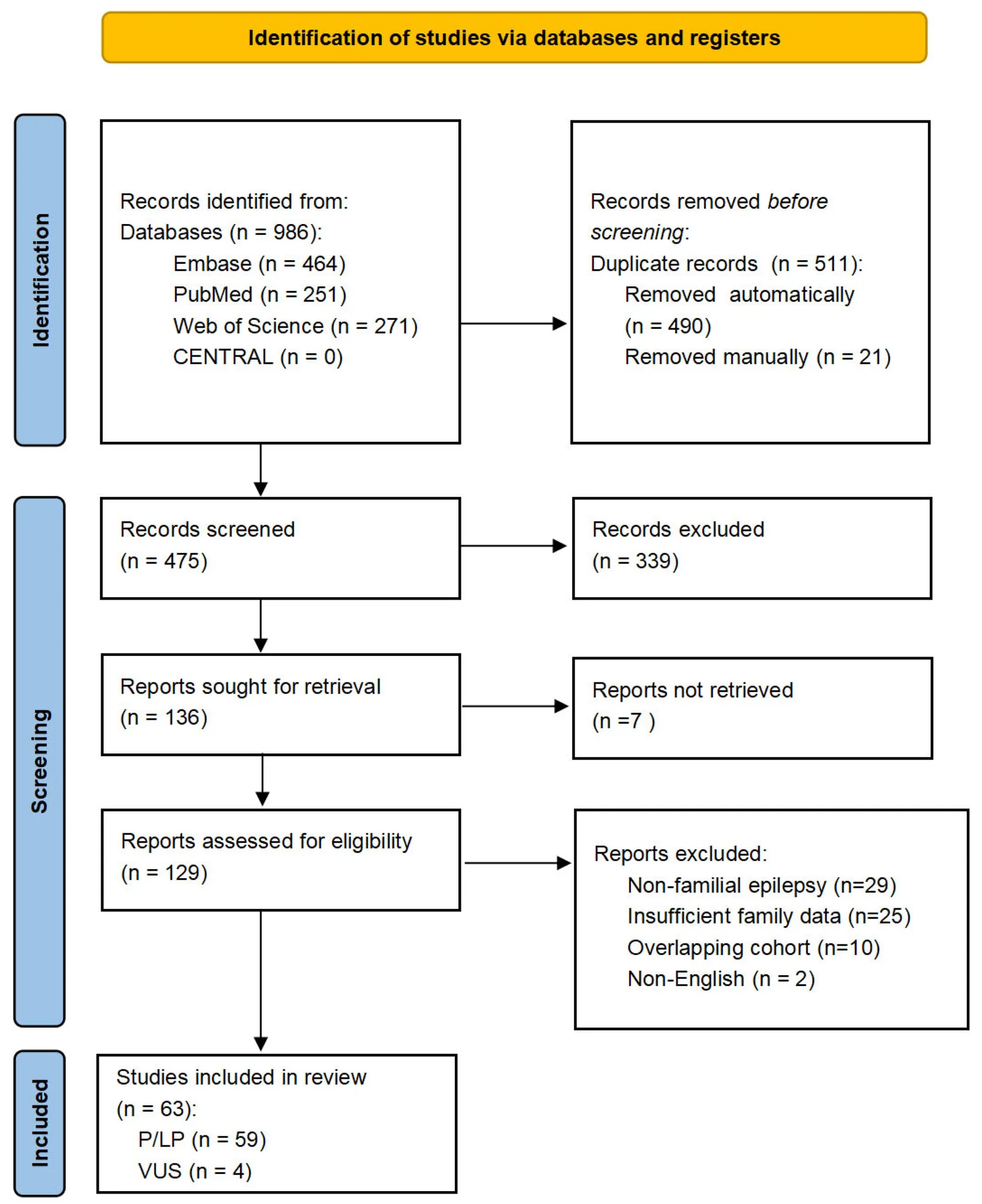
PRISMA Flow Diagram of Study Selection. P/LP, pathogenic or likely pathogenic; VUS, variants of uncertain significance.

This systematic review and meta-analysis was conducted in accordance with the Preferred Reporting Items for Systematic Reviews and Meta-Analyses (PRISMA) 2020 statement [17] and registered in the International Prospective Register of Systematic Reviews (PROSPERO: CRD420261388857). PubMed, Embase, CENTRAL, and Web of Science were searched from database inception to 15 June 2026. Search terms included “*DEPDC5*,” “*NPRL2*,” “*NPRL3*,” “GATOR1,” “epilepsy,” “seizure,” “focal cortical dysplasia (FCD),” “family,” “penetrance,” and “variant.” Complete database-specific search strategies are provided in Table S1. The study selection process is summarized in Figure 1.

### 2.2. Study Selection and Eligibility Criteria

Titles and abstracts were independently screened by two reviewers, followed by full-text assessment of potentially eligible articles. Disagreements were resolved through discussion or adjudication by a third reviewer.

Studies were included if they (1) were original human observational studies, case series, or family-based reports from which individual-level genotype–phenotype data could be reconstructed; (2) reported germline variants in *DEPDC5*, *NPRL2*, or *NPRL3* classified or re-evaluated as P/LP according to a GATOR1-specific classification framework [10], with detailed variant re-evaluation provided in Table S2; (3) included ≥2 individuals heterozygous for the familial variant per family, with ≥1 individual affected by epilepsy; and (4) represented the most comprehensive report when multiple publications described overlapping cohorts. Records for which familial variant status, epilepsy phenotype, or family membership could not be determined were excluded.

As epilepsy diagnostic criteria have evolved over time, confirmation of the epilepsy diagnosis according to the most recent International League Against Epilepsy (ILAE) classification was not required; diagnoses were accepted as reported by the source studies.

### 2.3. Data Extraction and Outcome Definitions

Two reviewers independently extracted pedigree structure, gene, variant consequence, recurrent variant status, parental origin, transmitting-parent phenotype, and, for *DEPDC5*, protein domain. Clinical variables included age at seizure onset, cognitive impairment, psychiatric/neurodevelopmental comorbidity, MRI findings, drug-resistant epilepsy (DRE), epilepsy surgery, histopathology, and postoperative seizure outcomes.

Variant consequences were classified as loss-of-function (LoF), in-frame indels, missense, or splice-region variants [10]. LoF variants included nonsense, frameshift, canonical splice-site variants, and copy-number variants. Recurrent variants were defined as identical variants reported in at least two unrelated families.

The primary outcome was the proportion of affected individuals among those heterozygous for germline P/LP variants in eligible pedigrees. Disease expression referred to the presence of epilepsy and was evaluated in all eligible individuals, whereas post-onset phenotype was evaluated only among affected individuals. Unaffected individuals were those heterozygous for germline P/LP variants who were reported not to have epilepsy at the time of assessment in the source study. Severity-related outcomes included age at seizure onset, cognitive impairment, psychiatric/neurodevelopmental comorbidity, MRI abnormalities, malformations of cortical development (MCD)/FCD, DRE, and epilepsy surgery. DRE and Engel classifications were recorded as reported in the source studies. Postoperative seizure outcomes were summarized descriptively.

### 2.4. Brain Somatic Second-Hit Synthesis

Brain somatic findings were analyzed separately from the primary family-based genotype–phenotype dataset. Somatic alterations reported among individuals in the primary dataset were first summarized, followed by an expanded synthesis of published cases in which a germline GATOR1 variant and an additional brain somatic alteration in the same GATOR1 gene were reported in the same individual. Duplicate cases were counted once. Somatic variant allele fractions (VAFs), sampled brain region, neuropathology, and paired brain–blood findings were extracted when available. Additional procedures for case reconciliation and multiregion VAF extraction are provided in Supplementary Methods S1.

### 2.5. Risk-of-Bias Assessment

Two reviewers independently assessed risk of bias using design-appropriate Joanna Briggs Institute critical appraisal tools [18,19,20] or the Newcastle–Ottawa Scale [21], as applicable. Detailed assessment procedures and criteria are provided in Supplementary Methods S2.

### 2.6. Statistical Analysis

Study-level proportions of affected individuals were pooled using an inverse-variance DerSimonian–Laird random-effects model applied to logit-transformed proportions and reported with 95% confidence intervals (CIs). A sensitivity analysis pooled family-level affected proportions using the same model. Heterogeneity was assessed using the *I*^2^ statistic and Cochran’s *Q* test. Small-study effects were evaluated using funnel plots and Egger’s test, with trim-and-fill analysis used to assess the robustness of the pooled estimate. The primary analysis was restricted to individuals heterozygous for P/LP variants; inclusion of variants of uncertain significance (VUS) was evaluated in a sensitivity analysis using the same meta-analytic approach.

Associations with disease expression and binary post-onset outcomes were analyzed using logistic regression with family-clustered robust standard errors. Prespecified factors included recurrent variant status, variant consequence, *DEPDC5* protein domain, parental origin, and transmitting-parent phenotype. Firth-penalized logistic regression or Fisher’s exact test was used when appropriate for sparse data. Age at seizure onset was summarized as median (interquartile range, IQR) and compared using Wilcoxon rank-sum or Kruskal–Wallis tests. Exploratory sensitivity analyses used family-cluster bootstrap CIs for median differences in age at seizure onset. For transmitting-parent phenotype, sensitivity analyses incorporated offspring proband status into models for onset age and six binary post-onset outcomes, while accounting for within-family correlation.

Missing data were not imputed, and analyses were based on available cases for each outcome. Subgroups with fewer than five individuals with available outcome data were summarized descriptively. Benjamini–Hochberg FDR correction was applied across the six binary post-onset outcomes separately within each comparison. Nominal *p*-values, denoted as *p*, were reported for disease-expression analyses, including parental-origin comparisons, and analyses of age at seizure onset. The six proband-adjusted binary-outcome analyses underwent a separate exploratory post hoc FDR correction. All tests were two-sided, with *p* < 0.05 or FDR-adjusted *p* < 0.05 considered statistically significant. Analyses were performed using R version 4.5.1 (R Foundation for Statistical Computing, Vienna, Austria) and GraphPad Prism version 10.0 (GraphPad Software, Boston, MA, USA). Additional statistical details are provided in Supplementary Methods S3.

## 3. Results

### 3.1. Study Characteristics

A total of 63 studies were included [2,3,4,8,9,10,11,22,23,24,25,26,27,28,29,30,31,32,33,34,35,36,37,38,39,40,41,42,43,44,45,46,47,48,49,50,51,52,53,54,55,56,57,58,59,60,61,62,63,64,65,66,67,68,69,70,71,72,73,74,75,76,77], of which 59 contributed to the primary meta-analysis [2,3,4,8,9,10,11,23,25,26,27,28,30,31,32,34,35,36,37,38,39,40,41,42,43,44,45,46,47,48,49,50,51,52,53,54,55,56,57,58,59,60,61,62,63,64,65,66,67,68,69,70,71,72,73,74,75,76,77]. Individual-level data were reconstructed for 734 individuals heterozygous for germline P/LP GATOR1 variants from 227 families, including 492 affected and 242 unaffected individuals (Table S3). Of these, 489 had *DEPDC5* variants, 41 had *NPRL2* variants, and 204 had *NPRL3* variants; the corresponding affected proportions were 66.3%, 65.9%, and 69.1%, respectively (Table S4).

**Figure 2:**
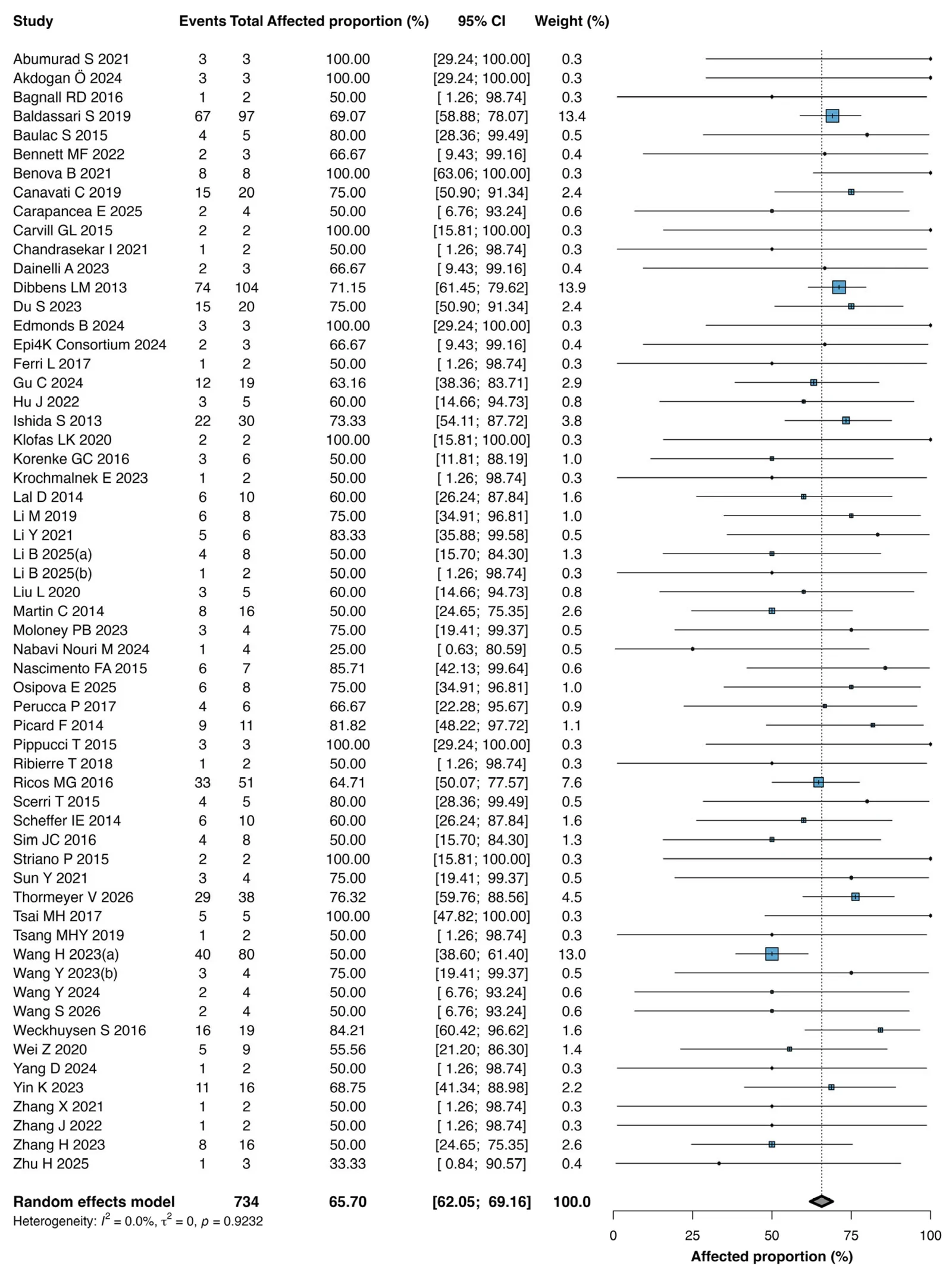
Forest plot of pooled proportions of affected individuals with germline GATOR1 P/LP variants. The studies included in the meta-analysis are cited in references [2,3,4,8,9,10,11,23,25,26,27,28,30,31,32,34,35,36,37,38,39,40,41,42,43,44,45,46,47,48,49,50,51,52,53,54,55,56,57,58,59,60,61,62,63,64,65,66,67,68,69,70,71,72,73,74,75,76,77]. Squares represent study-specific estimates, with their areas proportional to study weights. Horizontal lines indicate 95% CIs. The diamond represents the pooled estimate and its 95% CI. CI, confidence interval; P/LP, pathogenic or likely pathogenic.

The pooled proportion of affected individuals was 65.7% [95% CI, 62.05–69.16%] (Figure 2). Sensitivity analyses yielded similar estimates (Table S4), and the family-level pooled estimate was 63.6% (95% CI, 59.9–67.2%). Trim-and-fill adjustment had little effect on the pooled estimate (Figure S1). Risk-of-bias assessments are summarized in Table S5.

### 3.2. Familial Transmission

Across GATOR1, 316 matched adjacent-generation parent–offspring transmission pairs were analyzed. The proportions of affected offspring were 88.1% (104/118) among transmissions from affected parents and 93.4% (185/198) among transmissions from unaffected parents (*p* = 0.056) (Table S6). Proband offspring accounted for 54.2% (64/118) and 76.3% (151/198) of the respective groups, and all were affected.

**Figure 3:**
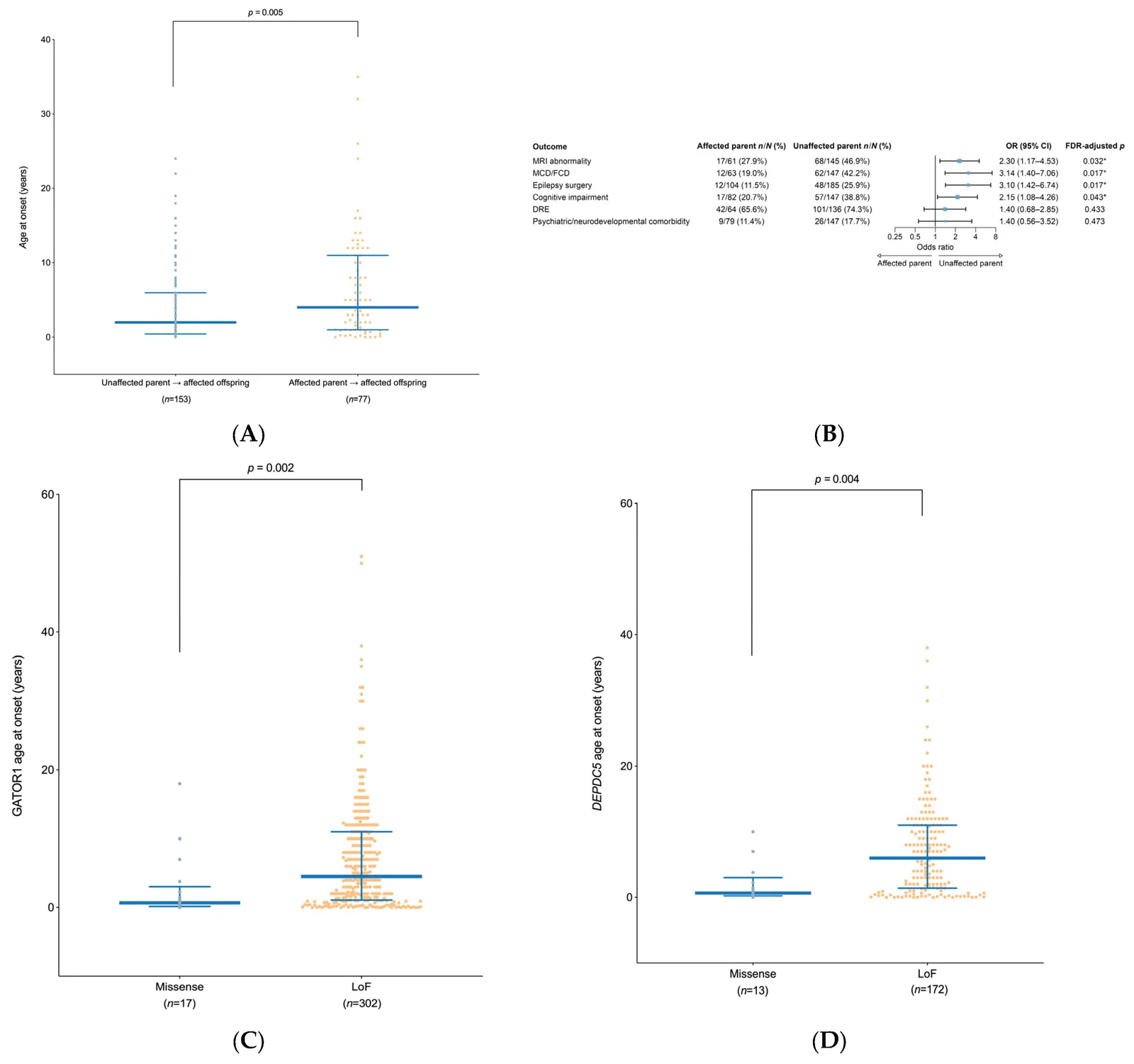
Familial and variant-level correlates of post-onset phenotype in GATOR1-related epilepsy. (**A**) Age at seizure onset according to transmitting-parent phenotype among affected offspring. (**B**) Clinical outcomes according to transmitting-parent phenotype in the primary analysis. ORs compare affected offspring of unaffected vs. affected transmitting parents. FDR-adjusted *p* < 0.05 (*). (**C**) Age at seizure onset in affected individuals with missense vs. LoF variants across GATOR1 genes. (**D**) Corresponding comparison restricted to *DEPDC5*. In panels (**A**,**C**,**D**), *n* indicates the number of affected individuals with available onset-age data, and *p*-values are nominal. Dots represent individual observations, with colors distinguishing the groups labeled on the x-axis; thick blue horizontal lines indicate medians, and blue error bars indicate interquartile ranges. OR, odds ratio; CI, confidence interval; MCD/FCD, malformation of cortical development/focal cortical dysplasia; DRE, drug-resistant epilepsy; LoF, loss-of-function.

Among affected offspring, those with an unaffected transmitting parent had an earlier median age at seizure onset than those with an affected transmitting parent [2.00 years (IQR 0.42–6.00; *n* = 153) vs. 4.00 years (IQR 1.00–11.00; *n* = 77); *p* = 0.005] (Figure 3A). The onset-age difference was statistically significant in the family-cluster bootstrap analysis (Table S7), but not in the proband-adjusted sensitivity analysis (Table S6).

Cognitive impairment was present in 38.8% (57/147) and 20.7% (17/82), respectively [odds ratio (OR) 2.15, 95% CI 1.08–4.26, FDR-adjusted *p* = 0.043]. The corresponding proportions were 46.9% (68/145) and 27.9% (17/61) for MRI abnormalities (OR 2.30, 95% CI 1.17–4.53, FDR-adjusted *p* = 0.032), 42.2% (62/147) and 19.0% (12/63) for MCD/FCD (OR 3.14, 95% CI 1.40–7.06, FDR-adjusted *p* = 0.017), and 25.9% (48/185) and 11.5% (12/104) for epilepsy surgery (OR 3.10, 95% CI 1.42–6.74, FDR-adjusted *p* = 0.017) (Figure 3B). Psychiatric/neurodevelopmental comorbidity and DRE did not differ significantly after FDR correction. In the proband-adjusted sensitivity analyses, none of the six binary outcomes was statistically significant after exploratory post hoc FDR correction (Table S6).

For GATOR1 overall, the affected proportions were 84.0% (178/212) after maternal transmission and 87.0% (181/208) after paternal transmission, with no significant difference between groups (OR 0.78, 95% CI 0.44–1.37, *p* = 0.385). Exploratory analysis within *DEPDC5* showed affected proportions of 82.4% (112/136) after maternal transmission and 90.4% (122/135) after paternal transmission (OR 0.50, 95% CI 0.26–0.94, *p* = 0.032). No significant differences were observed for *NPRL2* or *NPRL3* (Table S6).

### 3.3. Variant-Level Associations

For GATOR1 overall, individuals with recurrent variants had an affected proportion of 71.0% (154/217), compared with 65.4% (338/517) for non-recurrent variants (OR 1.32, 95% CI 0.98–1.78, *p* = 0.068). In affected individuals with available onset-age data, median age at seizure onset was 6.00 years (IQR 1.50–11.00, *n* = 93) for recurrent variants and 4.00 years (IQR 1.00–10.00, *n* = 230) for non-recurrent variants (*p* = 0.170) (Table S8). The onset-age difference was also not statistically significant in the family-cluster bootstrap analysis (Table S7).

The affected proportions across GATOR1 genes were 67.2% (427/635) for LoF variants, 70.0% (35/50) for in-frame indels, and 62.2% (28/45) for missense variants, with no significant difference among the three groups (*p* = 0.527). Of the four individuals with splice-region variants, two (50.0%) were affected.

In affected individuals with GATOR1 variants, missense variants were associated with an earlier median age at seizure onset than LoF variants [0.67 years (IQR 0.17–2.25, *n* = 17) and 4.50 years (IQR 1.14–10.95, *n* = 302), respectively; *p* = 0.002] (Figure 3C). A similar result was observed among individuals with *DEPDC5* variants [0.67 years (IQR 0.25–2.25, *n* = 13) and 6.00 years (IQR 1.40–11.00, *n* = 172), respectively; *p* = 0.004] (Figure 3D). Both onset-age differences remained statistically significant in family-cluster bootstrap analyses (Table S7). Median age at seizure onset was 5.00 years for in-frame indels and 2.09 years for splice-region variants, with data available for two affected individuals in each group.

For *DEPDC5* LoF variants, affected proportions across protein domains ranged from 50.0% to 70.2%. No significant differences relative to the N-terminal domain (NTD) were observed in domains with sufficient data for comparison. In analyses restricted to affected individuals, variants in the LassoA domain of DEPDC5, compared with NTD variants, were associated with higher frequencies of cognitive impairment [50.0% (11/22) and 17.1% (6/35), respectively; OR 4.83, 95% CI 1.48–15.80, FDR-adjusted *p* = 0.029], MRI abnormalities [55.0% (11/20) and 18.2% (4/22), respectively; OR 5.26, 95% CI 1.15–29.51, FDR-adjusted *p* = 0.046], and epilepsy surgery [24.2% (8/33) and 2.6% (1/38), respectively; OR 11.48, 95% CI 1.40–537.67, FDR-adjusted *p* = 0.029] (Table S9). Given the limited domain-specific sample sizes and wide CIs, these associations were considered exploratory.

### 3.4. Clinical and Surgical Features

The 492 affected individuals with GATOR1 variants had a median age at seizure onset of 4.00 years (IQR 1.00–10.00; *n* = 323), with gene-specific medians of 5.00 years for *DEPDC5*, 0.92 years for *NPRL2*, and 3.95 years for *NPRL3*. Cognitive impairment was reported in 23.4% (92/394), psychiatric/neurodevelopmental comorbidity in 13.6% (53/390), MRI abnormalities in 37.9% (99/261), and DRE in 63.8% (171/268).

Epilepsy surgery was performed in 13.3% (65/490). Histopathologic data were available for 54 individuals, with FCD IIa representing the predominant subtype (64.8% [35/54]). Of 50 individuals with paired MRI and histopathologic data, 8 (16.0%) had pathologically confirmed MCD/FCD despite negative MRI findings. Formal Engel classifications were available for 39 individuals, of whom 33 (84.6%) achieved Engel I–II outcomes (Table 1).

### 3.5. Brain Somatic Second Hits

Within the primary family-based genotype–phenotype dataset, brain somatic alterations were reported in four individuals. Three had a germline *DEPDC5* variant accompanied by a somatic alteration in the same gene, whereas one had a germline *NPRL3* variant and a somatic *WNT2* variant.

The expanded synthesis identified 14 published same-gene germline–somatic second-hit cases involving GATOR1 genes, including 13 *DEPDC5* and one *NPRL2* case (Table S10) [3,11,12,24,26,78,79,80,81,82,83,84,85,86]. FCD IIa was the most frequently reported histopathologic subtype among these cases, occurring in 12/14 cases (85.7%); the remaining two *DEPDC5* cases had FCD IIb and FCD I, respectively.

**Figure 4:**
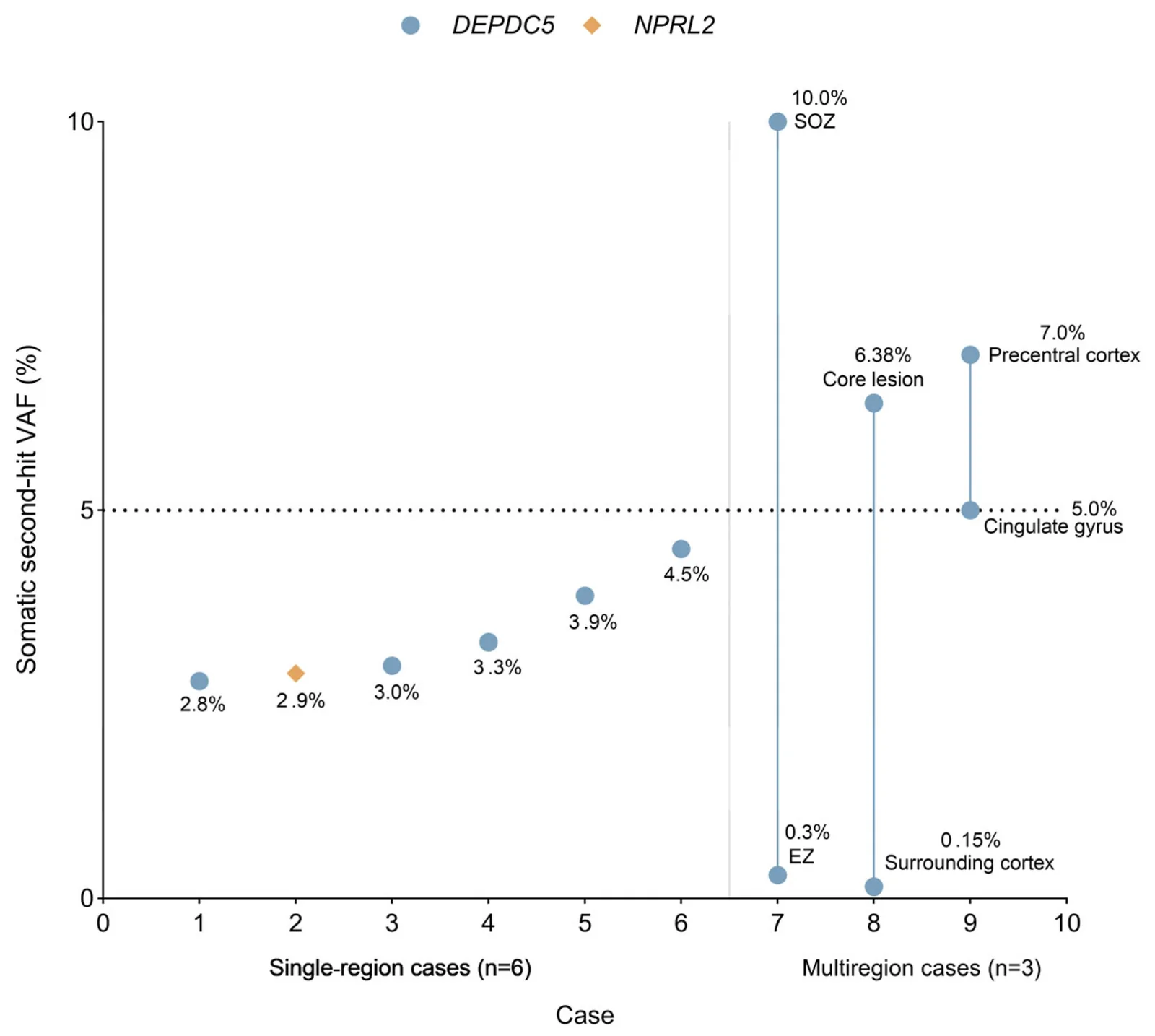
Brain somatic second-hit VAFs in GATOR1-related epilepsy. The horizontal dotted line indicates a VAF of 5%. The vertical light-gray line separates single-region from multiregion cases. Blue vertical lines connect samples obtained from different brain regions of the same individual, illustrating within-case spatial variation in somatic VAFs. EZ, epileptogenic zone; SOZ, seizure-onset zone; VAF, variant allele fraction.

Quantifiable somatic VAFs for sequence-variant second hits were available in nine cases. Six cases had single-region brain sampling, with VAFs ranging from 2.8% to 4.5%, all below 5% (Figure 4). Three *DEPDC5* cases underwent multiregion sampling, demonstrating spatial variation in somatic VAFs, with values as low as 0.15%. In two of these three cases, at least one sampled region had a VAF below 1%. In three cases with paired blood testing, the somatic event was detected in brain tissue but not in blood (Figure 4, Table S10).

## 4. Discussion

This systematic review and meta-analysis identified distinct association patterns for disease expression and post-onset phenotype in germline GATOR1-related epilepsy. Across *DEPDC5*, *NPRL2*, and *NPRL3*, the evaluated germline variant and familial characteristics showed no consistent associations with disease expression overall, whereas several associations emerged among affected individuals. Missense variants were associated with earlier seizure onset, and *DEPDC5* LassoA LoF variants showed exploratory associations with selected severity-related phenotypes. An exploratory paternal-transmission signal restricted to *DEPDC5* was also observed. Published same-gene germline–somatic second-hit cases predominantly involved *DEPDC5*, with FCD IIa the most frequently reported histopathologic subtype. Quantifiable second-hit VAFs were generally low, while multiregion sampling in three cases showed spatial variation.

Compared with previous *DEPDC5*-focused analyses [14], this study extended the assessment to all three GATOR1 genes. Reported variants were systematically re-evaluated using GATOR1-specific classification criteria [10], and the primary analysis was restricted to individuals heterozygous for germline P/LP variants. Reconstructed pedigrees and matched parent–offspring pairs enabled evaluation of transmitting-parent phenotype and parental origin. Brain somatic second-hit evidence added a complementary tissue-level perspective.

Reduced penetrance and phenotypic variability are well recognized in GATOR1-related epilepsy [10], but the relationship between parental phenotype and offspring outcomes remains unclear. Transmitting-parent phenotype was not significantly associated with offspring disease expression, yet affected offspring of unaffected parents had earlier seizure onset and higher frequencies of cognitive impairment, MRI abnormalities, MCD/FCD, and epilepsy surgery in the primary analyses. The proband-adjusted sensitivity analyses provided weaker support for these associations, which may partly reflect ascertainment differences. These exploratory associations do not establish transmitting-parent phenotype as a predictor of offspring clinical severity.

An exploratory gene-specific signal also emerged from parental origin. At the overall GATOR1 level, disease expression was similar after paternal and maternal transmission, whereas within *DEPDC5*, paternal transmission was associated with a higher proportion of affected offspring. Current data do not establish imprinting or another defined parent-of-origin mechanism; rather, the *DEPDC5*-specific signal should be considered hypothesis-generating and warrants evaluation in larger independent familial datasets.

Affected individuals with missense variants had earlier seizure onset than those with LoF variants, both across GATOR1 and within *DEPDC5*. Given the small number of affected individuals with missense variants, this association should be interpreted cautiously. Functional studies of *DEPDC5* variants have shown diverse effects on RNA processing, including abnormal splicing caused by some missense variants [43]. *DEPDC5* haploinsufficiency has been shown to increase mTORC1 signaling and alter neuronal morphology in human iPSC-derived cortical neurons [34]. Whether altered splicing or other variant-specific functional effects contribute to earlier seizure onset requires further investigation.

Previous research has suggested molecular subregional effects of *DEPDC5* variants, particularly in relation to malformations of cortical development [39]. In the present analysis restricted to LoF variants, domain location did not clearly distinguish disease expression, whereas LassoA variants were associated with higher frequencies of cognitive impairment, MRI abnormalities, and epilepsy surgery among affected individuals. LassoA lies adjacent to the SHEN domain, which contributes to the interface between DEPDC5 and Rag GTPases [6]. LoF variants in this region that generate truncated proteins could therefore affect downstream SHEN-related structure. The potential relevance of this interface is supported by evidence that brain-enriched RagB isoforms modulate mTORC1 dynamics through GATOR1 inhibition involving DEPDC5 [87]. These exploratory LassoA associations warrant further validation.

The clinical findings were consistent with the recognized spectrum of focal epilepsy and cortical malformations in GATOR1-related disease [9]. In our synthesis, MCD/FCD was identified histopathologically in some individuals despite negative MRI findings. Bottom-of-sulcus dysplasia and related focal cortical lesions can be radiologically subtle and may remain unrecognized on conventional MRI despite well-localized focal epilepsy [80]. These findings support detailed imaging reassessment and multimodal presurgical localization in individuals with germline GATOR1 variants when electroclinical findings indicate a focal origin despite negative MRI. Favorable postoperative seizure outcomes also support epilepsy surgery as a treatment option in appropriately selected individuals, consistent with previous reports [88].

Brain somatic second hits provide a mechanistic link between germline GATOR1 susceptibility and focal cortical pathology. FCD IIa predominated in the published same-gene second-hit cases, consistent with previous reports of GATOR1-related FCD [10]. Germline–somatic biallelic disruption has been demonstrated in *DEPDC5*-related FCD, with somatic events in some cases restricted to dysmorphic neurons [11,12]. Functional support comes from cortical organoid models, in which mosaic biallelic DEPDC5 loss increases mTOR activity, alters neuronal morphology, and enhances excitability [89]. The three cases with multiregion sampling in our synthesis exhibited spatial variation in second-hit VAFs. Such variation parallels the regional differences in mutation burden and dysmorphic-neuron density described previously [11,12]. A molecular spatial continuum offers a possible interpretation, although this hypothesis requires further investigation. Beyond mutation-bearing cells, single-cell analyses have identified transcriptional abnormalities in nonmutated FCD II cells [84]. Experimental studies also show that astrocytic GATOR1 dysfunction impairs mitochondrial metabolism and alters glutamate–glutamine cycling [90]. Phenotypic variability in GATOR1-related epilepsy may therefore reflect inherited susceptibility modified by developmental, cellular, and brain-specific factors rather than germline genotype alone.

Several limitations should be considered. Most source data were derived from published pedigrees, retrospective series, and referral-center cohorts that were commonly ascertained through affected probands. This may introduce selection and publication bias, while incomplete testing of unaffected relatives may overestimate the affected proportion and influence analyses of transmitting-parent phenotype and parental origin. The pooled estimate should therefore not be generalized to unselected individuals with GATOR1 variants. Affected proportions in selected transmission pairs should neither be directly compared with this estimate nor interpreted as prospective offspring risks. Phenotype definitions, follow-up duration, imaging protocols, and genetic testing methods varied across studies, and missing data resulted in outcome-specific denominators. Gene-specific, transmission-related, and domain-level analyses involved smaller samples, with sparse events contributing to wide confidence intervals for some estimates, particularly the LassoA findings. Direct functional characterization was unavailable for most individual variants, limiting mechanistic interpretation of variant-consequence and domain-related associations. Brain somatic second-hit analyses were additionally subject to case-selection, tissue-sampling, and technical constraints. Brain tissue was obtained almost exclusively from individuals undergoing epilepsy surgery, so the observed pathological distribution may not represent all individuals with GATOR1 variants. Cellular and spatial mosaicism within FCD lesions, together with differences in tissue sampling and sequencing methods, may affect second-hit detection. Low-level somatic variants may fall below detection thresholds, while copy-number changes, loss of heterozygosity, and other structural second-hit events may also be missed. Accordingly, the available data cannot establish the true prevalence of brain somatic second hits among all individuals heterozygous for germline GATOR1 variants. Failure to detect a second hit does not exclude this mechanism.

## 5. Conclusions

Familial transmission highlighted intrafamilial phenotypic variability, although associations involving transmitting-parent phenotype remain exploratory. Exploratory associations were observed between missense variants and earlier seizure onset, and between DEPDC5 protein location and selected severity-related phenotypes. Published second-hit cases further support a role for brain-specific somatic variation in focal cortical pathology. Separate assessment of disease expression and post-onset phenotype may help explain clinical heterogeneity in GATOR1-related epilepsy.

## Figures and Tables

**Table 1:** Clinical characteristics and outcomes among affected individuals with GATOR1-related epilepsy.

Characteristic	Overall (*N* = 492)	*DEPDC5* (*N* = 324)	*NPRL2* (*N* = 27)	*NPRL3* (*N* = 141)
Clinical characteristics				
Age at seizure onset, years, median (IQR) [*n*]	4.00 (1.00–10.00) [*n* = 323]	5.00 (1.00–10.00) [*n* = 189]	0.92 (0.07–6.00) [*n* = 16]	3.95 (1.33–10.00) [*n* = 118]
Cognitive impairment, *n*/*N* (%)	92/394 (23.4%)	64/247 (25.9%)	8/26 (30.8%)	20/121 (16.5%)
Psychiatric/neurodevelopmental comorbidity, *n*/*N* (%)	53/390 (13.6%)	45/246 (18.3%)	1/26 (3.8%)	7/118 (5.9%)
Neuroimaging and epilepsy outcomes				
MRI abnormality, *n*/*N* (%)	99/261 (37.9%)	59/158 (37.3%)	7/13 (53.8%)	33/90 (36.7%)
DRE, *n*/*N* (%)	171/268 (63.8%)	103/156 (66.0%)	11/16 (68.8%)	57/96 (59.4%)
Epilepsy surgery, *n*/*N* (%)	65/490 (13.3%)	35/324 (10.8%)	7/26 (26.9%)	23/140 (16.4%)
Histopathology available, *n*	54	31	7	16
FCD I, *n* (%)	10 (18.5%)	4 (12.9%)	1 (14.3%)	5 (31.2%)
FCD IIa, *n* (%)	35 (64.8%)	20 (64.5%)	5 (71.4%)	10 (62.5%)
FCD IIb, *n* (%)	2 (3.7%)	2 (6.5%)	0 (0.0%)	0 (0.0%)
mMCD, *n* (%)	5 (9.3%)	4 (12.9%)	1 (14.3%)	0 (0.0%)
Other, *n* (%)	2 (3.7%)	1 (3.2%)	0 (0.0%)	1 (6.2%)
Engel classification available, *n*	39	23	5	11
Engel I, *n* (%)	31 (79.5%)	20 (87.0%)	4 (80.0%)	7 (63.6%)
Engel II, *n* (%)	2 (5.1%)	2 (8.7%)	0 (0.0%)	0 (0.0%)
Engel III–IV, *n* (%)	6 (15.4%)	1 (4.3%)	1 (20.0%)	4 (36.4%)

Percentages were calculated using the number of individuals with reported data for each outcome. Engel classifications were extracted as reported in the source studies: class I, freedom from disabling seizures; class II, rare disabling seizures; class III, worthwhile improvement; class IV, no worthwhile improvement. DRE, drug-resistant epilepsy; FCD, focal cortical dysplasia; mMCD, mild malformation of cortical development; IQR, interquartile range.

## Data Availability

No new primary data were generated in this study. Data extracted from published studies and used in the analyses are provided in the article and its Supplementary Materials.

## Supplementary Materials

The following supporting information can be downloaded at https://www.mdpi.com/article/10.3390/ijms27219773/s1.

## Abbreviations

The following abbreviations are used in this manuscript:

CI	Confidence interval
DRE	Drug-resistant epilepsy
FCD	Focal cortical dysplasia
ILAE	International League Against Epilepsy
IQR	Interquartile range
LoF	Loss-of-function
MCD	Malformation of cortical development
NTD	N-terminal domain
OR	Odds ratio
P/LP	Pathogenic or likely pathogenic
VAF	Variant allele fraction
VUS	Variant of uncertain significance
