# Supplementary material for: Familial Transmission and Phenotypic Heterogeneity in Germline GATOR1-Related Epilepsy: A Systematic Review and Meta-Analysis
Source: Int J Mol Sci. 2026 Oct 30;27(21):9773. doi: 10.3390/ijms27219773 (PMC13763648; doi:10.3390/ijms27219773)
Supplement: Supplementary file 1 [file ijms-27-09773-s001.zip › Supplementary materials-ijms-4562790/Supplementary Methods and Figures-ijms-4562790.pdf]

Familial Transmission and Phenotypic Heterogeneity in Germline GATOR1-Related Epilepsy: A Systematic Review and Meta-Analysis

Contents

Supplementary Methods

Supplementary Methods S1. Brain Somatic Second-Hit Data Extraction

Supplementary Methods S2. Risk-of-Bias Assessment

Supplementary Methods S3. Additional Statistical Details

Supplementary Figures

Supplementary Figure S1. Funnel Plot for the Meta-Analysis of Affected Proportions

Supplementary Methods S1. Brain Somatic Second-Hit Data Extraction

Brain somatic findings were evaluated separately because brain-tissue testing was not required for inclusion in the primary family-based analysis. The primary dataset included somatic findings reported among otherwise eligible individuals, whereas the expanded synthesis additionally included published GATOR1 cases meeting the predefined same-gene germline–somatic second-hit criterion. When multiple publications described the same individual, the case was counted once and information was reconciled across reports. Extracted variables included germline and somatic alterations, sampled brain region, neuropathology, somatic variant allele fraction (VAF), and paired brain–blood findings when available. For multiregion sampling, region-specific VAFs were retained rather than collapsed into a single summary value to characterize spatial heterogeneity.

Supplementary Methods S2. Risk-of-Bias Assessment

Two reviewers independently assessed the risk of bias of the included studies using design-specific appraisal tools. Disagreements were resolved through discussion or adjudication by a third reviewer. Case reports and case series or family series were evaluated using the corresponding Joanna Briggs Institute (JBI) critical appraisal tools [18,19]. Eligible analytical cross-sectional studies were assessed using the revised 2026 JBI tool [20]. Cohort and case-control studies were evaluated using the Newcastle–Ottawa Scale (NOS) [21].

JBI items were rated as “Yes,” “No,” “Unclear,” or “Not applicable.” Overall judgments were based on the pattern and methodological importance of the assessed domains rather than on numerical summary scores. Studies were judged to have a low risk of bias when the core domains of case definition, participant selection, genetic and clinical ascertainment, and outcome assessment were adequately addressed. Moderate risk was assigned when core ascertainment was acceptable but one or more noncritical domains were unclear. High risk was assigned when a critical limitation was considered likely to materially affect the findings.

For the NOS, cohort studies were evaluated across selection, comparability, and outcome domains, whereas case-control studies were evaluated across selection, comparability, and exposure domains. Scores of 7–9, 4–6, and 0–3 stars were prespecified as indicating low, moderate, and high risk of bias, respectively. Study-level assessments are presented in Table S5.

Supplementary Methods S3. Additional Statistical Details

For the meta-analysis of affected proportions, a continuity correction of 0.5 was applied only to studies with zero or all events. Funnel plots, Egger's test, and trim-and-fill analysis were used to assess potential small-study effects when a sufficient number of studies were available.

ACMG classifications and supporting criteria were extracted as reported in the source studies, where available, and are presented alongside the GATOR1-specific re-evaluation in Table S2.

For genotype–phenotype analyses, *DEPDC5* protein domains were compared with the N-terminal domain (NTD) as the reference category. Family-clustered robust standard errors were used to account for within-family correlation. Firth penalized logistic regression was applied when conventional logistic regression showed non-convergence, sparse data, or possible separation. Sparse non-zero 2×2 tables were evaluated using Fisher's exact test. Subgroups with fewer than five individuals with available outcome data and comparisons for which stable inferential estimates could not be obtained were summarized descriptively.

Exploratory sensitivity analyses of onset age by recurrent variant status, variant consequence, and transmitting-parent phenotype used 10,000 bootstrap resamples of entire families to estimate percentile 95% CIs for median differences. For transmitting-parent phenotype, additional exploratory sensitivity analyses adjusted for offspring proband status using median regression with 10,000 family-cluster wild gradient bootstrap replicates. The 95% CIs and two-sided p-values were calculated using bootstrap standard errors and a Wald t approximation.

Missing or unreported clinical features were not classified as absent. Individuals with missing data for a specific outcome were excluded only from the corresponding analysis, resulting in outcome-specific denominators. Missing values were not imputed.

For the six binary post-onset outcomes, separate Benjamini–Hochberg FDR correction families were defined for transmitting-parent phenotype, recurrent versus non-recurrent variants, each variant-consequence category versus LoF, and each DEPDC5 domain versus NTD. Where analyses were stratified by gene or variant consequence, correction was performed separately within each stratum and comparison.

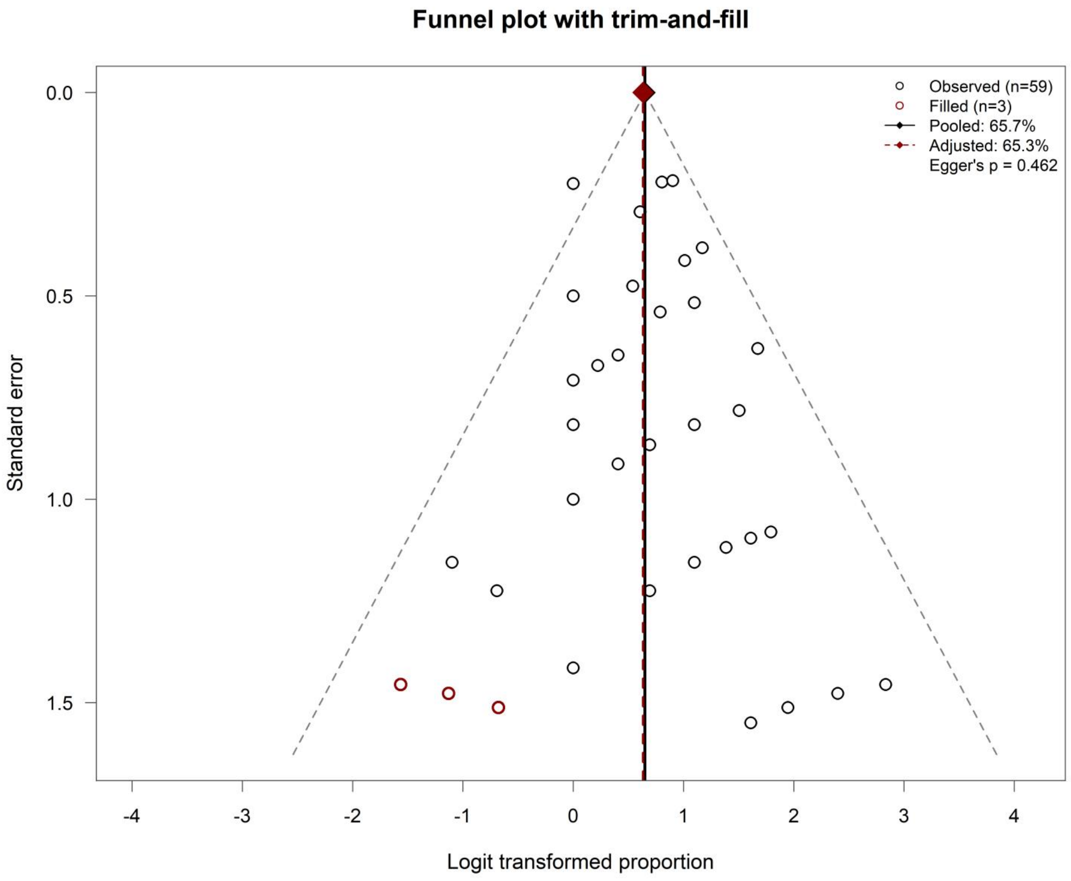

Supplementary Figure S1. Funnel Plot for the Meta-Analysis of Affected Proportions
